# Engineered Peptide Scrambling for Enhanced Drug Delivery to Resistant Breast Cancer Cells via Small Extracellular Vesicles

**DOI:** 10.1021/acsabm.5c00582

**Published:** 2025-12-01

**Authors:** Anika Babel, Joe Yuan, Najla A Saleh, Aimen Al-Hilfi, Sadhana Kilangodi, Jake Sun, Lelti Asgedom, Alicia Withrow, Assaf A. Gilad, Masamitsu Kanada

**Affiliations:** † Institute for Quantitative Health Science and Engineering (IQ), 3078 Michigan State University, East Lansing, Michigan 48824, United States; ‡ College of Natural Science, Michigan State University, East Lansing, Michigan 48824, United States; § Department of Chemical Engineering & Materials Science, Michigan State University, East Lansing, Michigan 48824, United States; ∥ College of Engineering, Michigan State University, East Lansing, Michigan 48824, United States; ⊥ College of Osteopathic Medicine, Michigan State University, East Lansing, Michigan 48824, United States; # Center for Advanced Microscopy, Michigan State University, East Lansing, Michigan 48824, United States; ∇ Department of Radiology, Michigan State University, East Lansing, Michigan 48824, United States; ○ College of Human Medicine, Michigan State University, East Lansing, Michigan 48824, United States; ◆ The Scojen Institute for Synthetic Biology, Reichman University, Herzliya, 4610101, Israel; ¶ Department of Pharmacology & Toxicology, Michigan State University, East Lansing, Michigan 48824, United States

**Keywords:** extracellular vesicle, tumor homing peptide, methotrexate (MTX), cancer, bioluminescence, fluorescence, chemotherapy

## Abstract

Extracellular vesicle (EV)-mediated transfer of biomolecules plays an essential role in intercellular communication and presents promising avenues for targeted drug delivery. Over the past decade, researchers have developed various approaches to modifying EV surfaces for targeting specific cells or tissues, including functionalization with targeting peptides to increase the specificity of drug delivery. Due to technical limitations, methods for characterizing the targeting moieties on the surface of small EVs (sEVs) are considerably restricted. To address these limitations and enhance the throughput capacity of sEV characterization, a dual-reporter platform was utilized to quantitatively assess the binding of tumor homing peptide (THP)-functionalized sEVs to breast cancer cells using bioluminescence assays and fluorescence microscopy. Twenty-four scrambled variants of the uPAR-binding peptide were designed for sEV engineering, and their uptake by MDA-MB-231 cells was evaluated in vitro. Our results revealed that amino acid scrambling generated both enhanced and reduced binding to cancer cells compared to the original peptide sequence. Furthermore, the data demonstrated that mechanical stimulation of EV producer HEK293FT cells enhanced the passive loading of methotrexate (MTX) into sEVs, but not large EVs, by increasing sEV production. By functionalizing MTX-loaded sEVs with a high-binding scrambled peptide, the delivery successfully surpassed the saturated free MTX uptake level in MDA-MB-231 cells, increasing cytotoxicity by 2.1-fold and providing a potent strategy for combating drug-resistant cancers. This study advances synthetic biology approaches to optimize tumor-targeted drug delivery, demonstrating that strategic peptide sequence scrambling can enhance targeting efficiency and drug delivery capabilities.

## Graphical abstract

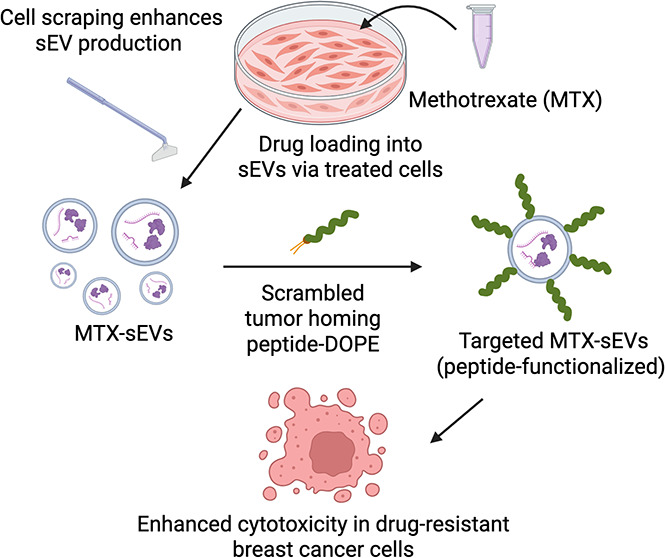

## Introduction

Bioactive peptides represent a significant therapeutic class, with nearly 100 peptide-based drugs approved worldwide since insulin therapy began in 1922. The peptide therapeutics market continues to grow with ongoing clinical development.^1^ Applications have expanded from hormone replacement to treatments for blood pressure, coagulation, and pain management.^2^ Projections indicate substantial growth for the therapeutic peptide market in the coming year, notably driven by breakthrough drugs such as Tirzepatide and Semaglutide, which have revolutionized obesity management and significantly increased the market value of peptide therapeutics.^3^ Peptides are defined as short amino acid chains that exhibit diverse protein-like functions; many of which are derived from naturally occurring protein fragments, such as cell-penetrating peptides (e.g., HIV-1 Tat and penetratin from *Drosophila* Antennapedia).^4–6^ While peptides offer advantages over proteins including easier synthesis, enhanced storage stability, and simplified handling, they face limitations such as restricted target specificity, short serum half-life, and susceptibility to proteolytic degradation.^7^ Despite these drawbacks, advancements in synthetic and recombinant technologies have progressively augmented the utility of bioactive peptides in therapeutic and diagnostic applications. Furthermore, the integration of artificial intelligence methodologies accelerates the development of novel peptides with exceptional properties.^8–11^

The field of cancer treatment has witnessed significant advancement through the development of nanomaterials capable of precisely targeting malignant cells while sparing healthy tissue.^12^ Synthetic peptides serve as promising scaffolds for creating such materials, but still present substantial challenges when designing peptides for targeted drug delivery. Specifically, there remains a lack of established methodologies to accurately predict how particular amino acid sequences influence peptide folding, self-assembly behaviors, and structural stability. Consequently, our understanding of how to strategically engineer these molecular properties to exploit unique cancer cell features—such as altered pH environments, overexpressed receptors, or dysregulated metabolic pathways—remains limited.^13,14^ This knowledge gap hinders the rational design of peptides that can effectively differentiate between healthy and malignant tissues based on their distinct physiological signatures.

Cell-derived extracellular vesicles (EVs), which play crucial roles in intercellular communication, have emerged as promising nanocarriers. All cells in the body produce EVs, including apoptotic bodies (>1 μm in diameter), ectosomes or microvesicles (100 nm – 1 μm in diameter), and exosomes (30–200 nm in diameter).^15,16^ EVs exhibit remarkable biocompatibility and possess the capacity to protect and deliver therapeutic macromolecules (e.g., DNA, mRNA, and miRNA) to target cells and tissues. However, despite encouraging advancements,^17^ systemically administered EVs are rapidly eliminated by immune cells, exhibiting a short half-life of ∼ 2 h.^18,19^ This rapid clearance results in nonspecific tissue accumulation, limiting their therapeutic potential. Consequently, targeting modifications are imperative to improve efficiency and minimize off-target effects. To date, several studies have demonstrated peptide-based strategies for directing EVs toward tumor cells.^20^ For example, EVs were targeted to tumor cells by fusing Lamp2b with the integrin-specific peptide iRGD. These engineered EVs selectively delivered doxorubicin to tumors, inhibiting growth while sparing non-neoplastic cells.^21^ Similarly, Ohno et al. modified EVs with GE11 peptide to target EGFR-overexpressing cancer cells. These EVs delivered let-7a miRNA specifically to xenograft breast cancer mouse models, demonstrating potential for miRNA replacement cancer therapies.^22^ It is important to acknowledge, however, that surface modification of EVs with peptides may significantly affect the molecular composition of the protein corona, which could influence cellular uptake and biodistribution.^23^ The absorption of dysopsonins, such as albumin and apolipoproteins, prolongs circulation time, while opsonins, including immunoglobulins and complement proteins, lead to rapid immune-mediated clearance.^24^ Further investigations are needed to understand the interaction mechanisms between plasma proteins and peptide-functionalized EVs to enable rational design of EVs with enhanced targeting efficiency and reduced off-target accumulation.

Previously, we developed a dual-reporter platform to characterize the cellular uptake of tumor-homing peptide (THP)-functionalized sEVs, and identified the urokinase-type plasminogen activator receptor (uPAR)-binding peptide as exhibiting the highest binding affinity to MDA-MB-231 breast cancer cells, which overexpress uPAR.^25^ While peptides are easily designed and manipulated, controlling their folding structures and functions remains challenging, primarily attributed to their intrinsically disordered folding patterns.^26^ In the current study, we investigated the impact of amino acid scrambling of the THP on functionalized sEVs. Intriguingly, this scrambling resulted in superior binding to cancer cells for some variants, while others showed diminished function. These high-binding variants enhanced the efficacy of intracellular delivery of therapeutic molecules, thereby highlighting an unrecognized importance of amino acid patterns within peptides for optimizing potent THPs for targeted drug delivery via sEVs.

## Results and Discussion

### Dual Bioluminescent-Fuorescent Reporter PalmReNL for Bulk sEV Quantification and Single-sEV Tracking

We previously demonstrated that the palmitoylated red-shifted BRET EV reporter, PalmReNL, enables quantitative tracking of sEVs both in vitro and in vivo.^19^ In this study, we engineered HEK293FT cells to stably express PalmReNL and isolated sEVs from conditioned medium using 50 nm membrane filters.^27,28^ Nanoparticle Tracking Analysis (NTA) revealed that PalmReNL-containing sEVs had a concentration of 4.4 × 10^10^ EVs/mL, with peak and mean diameters of 95 and 120.1 nm, respectively (Figure S1A). In comparison, sEVs from unmodified HEK293FT cells had a concentration of 7.0 × 10^9^ EVs/mL, with peak and mean diameters of 110.5 and 129.4 nm, respectively. These results indicate that PalmReNL expression does not significantly impact sEV production or size distribution. Western blot analysis further confirmed that PalmReNL expression did not alter the levels of sEV markers CD63 and CD81 (Figure S1B). Bioluminescence measurements using 25 μM furimazine showed a linear relationship between PalmReNL-sEV concentration and bioluminescence intensity, supporting the use of PalmReNL for quantitative sEV assessment (Figure S1C). Additionally, the tdTomato component of PalmReNL enabled fluorescence-based visualization of individual sEVs, as we previously demonstrated^19,29^ (Figure S1D). It is important to note, however, that conventional fluorescence microscopy cannot accurately resolve individual sEVs due to their subdiffraction-limited size (∼250 nm). We previously demonstrated that fluorescence signals from PalmReNL-sEVs exhibited poor linearity compared to the highly linear bioluminescence signals from the same dual-reporter sEVs.^25^ These complementary characteristics of the dual-reporter system highlight the distinct advantages of quantitative bioluminescence measurements and qualitative fluorescence visualization for comprehensive sEV characterization.

### Surface Modification of sEV with Scrambled THPs Resulted in Enhanced Uptake

We previously utilized PalmReNL-sEVs to evaluate several THPs for sEV surface modification to enhance tumor targeting capacity. Among these, sEVs functionalized with DOPE-conjugated uPAR-binding peptides (VSNKYFSNIHWGC) demonstrated the highest uptake in MDA-MB-231 breast cancer cells, which exhibit high uPAR protein expression.^25^ In the current study, we created 24 scrambled variants of the uPAR-binding peptide, maintaining the 13-residue composition while randomized amino acid positions. Subsequently, we screened the uptake efficiency of sEVs functionalized with DOPE-conjugated scrambled uPAR-binding peptides in MDA-MB-231 cells. Additionally, we examined PalmReNL-sEVs modified with nine nonspecific 13-mer peptides conjugated with DOPE to assess nonspecific uptake under our experimental conditions as a control (Figure S2). PalmReNL-sEVs derived from HEK293FT cells were engineered with either 24 scrambled or nine nonspecific 13-mer peptides. These peptide-engineered PalmReNL-sEVs were incubated with MDA-MB-231 cells for 3 h, and cellular uptake was evaluated by measuring bioluminescence (Figure 1A).

**Figure 1:**
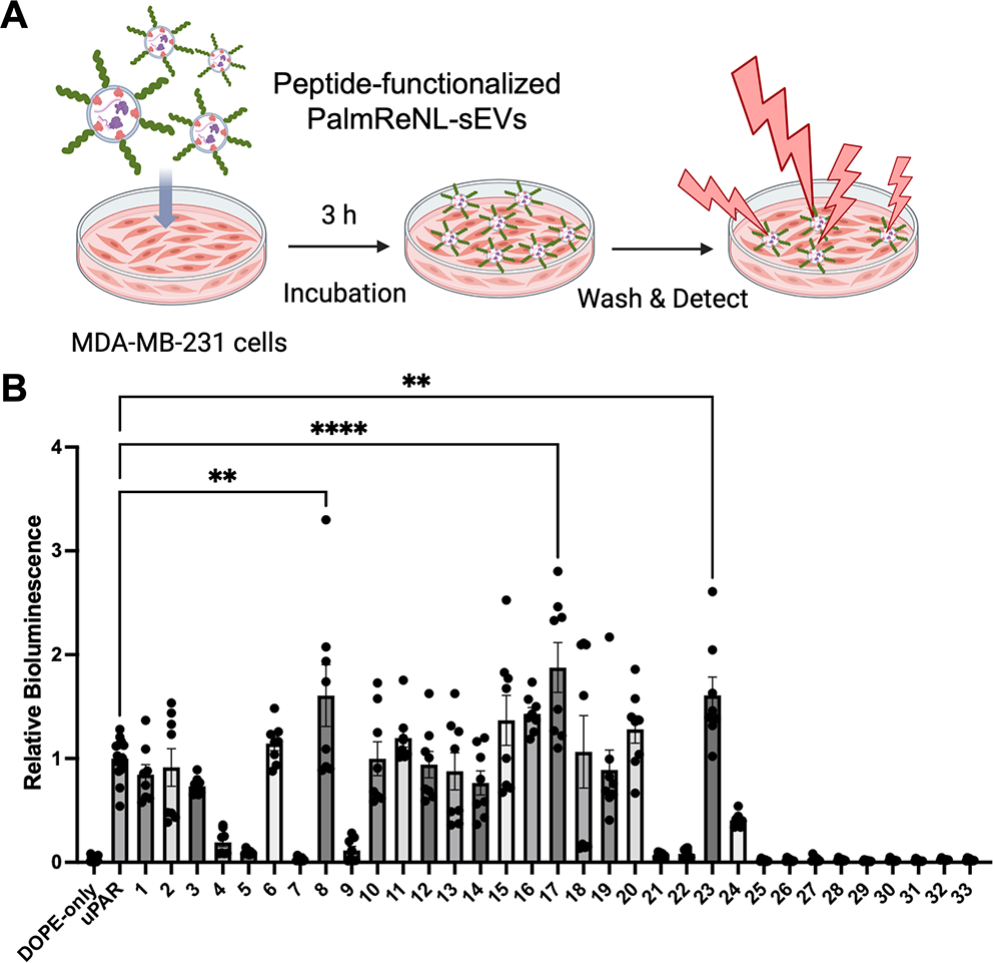
Screening scrambled THPs to optimize sEV-based tumor-targeted drug delivery. (A) Schematic representation of the assay for cancer cell uptake of engineered PalmReNL-labeled sEVs (Created with BioRender.com). (B) Cellular uptake of PalmReNL-sEVs was analyzed by measuring bioluminescence in MDA-MB-231 cells following treatment with sEVs displaying various peptide variants. Cells (40,000/well) were treated with PalmReNL-sEVs (3.8 × 10^8^ particles/well) for 3 h. DOPE-only PalmReNL-sEVs (negative control) were engineered with the DOPE probe but no peptides. uPAR PalmReNL-sEVs (positive control) displayed the original uPAR-binding peptide sequence. Samples 1–24 represent PalmReNL-sEVs engineered with different scrambled uPAR-binding peptides, while samples 25–33 contain 13-mer nonspecific peptides. Error bars represent SD (*n* = 8).

Interestingly, while scrambled peptides commonly serve as negative controls, three scrambled peptides (#8, #17, and #23) enhanced sEV uptake by 1.6-, 1.9-, and 1.6-fold, respectively, compared to the original uPAR-binding peptide (Figure 1B). In contrast, we found that only six of the 24 scrambled peptides (#4, #5, #7, #9, #21, and #22) were suitable as negative controls for the original uPAR-binding peptides. These findings suggest that specific amino acid positions exert a significant influence on cellular binding capacities, despite identical amino acid compositions, net charge, and hydrophobicity across the peptides (Figure S2). Notably, all nonspecific 13-mer peptides displayed reduced EV uptake compared to the uPAR-binding peptide, confirming their lack of targeting efficacy for cancer cells during the 3-h incubation period. We further evaluated the cellular uptake of PalmReNL-sEVs functionalized with Peptides 8, 17, 23, alongside the original uPAR-binding peptide, across a range of concentrations to determine their dissociation constants (*K*_d_). The high-binding peptides (#8, #17, and #23) enhanced cancer cell uptake by 2.5-, 5.2-, and 3.5-fold, respectively, compared to the original uPAR-binding peptide (Figure S3).

### Characterization of Individual Cancer Cell Uptake of Peptide-Engineered PalmReNL-sEVs Using Fluorescence Microscopy

We further characterized the cellular uptake of peptide-functionalized PalmReNL-sEVs by directly visualizing tdTomato protein within PalmReNL using fluorescence microscopy. MDA-MB-231 cells were incubated with PalmReNL-sEVs bearing different scrambled THPs for 3 h, followed by staining with CellTrace CFSE for fluorescence microscopy analysis. We compared sEVs engineered with peptides #8, #17, and #23, which enhanced binding to cancer cells, with sEVs bearing peptides #7, #21, and #22, which reduced cancer cell uptake (Figures 2 and S4). Fluorescence microscopy revealed that PalmReNL-sEVs bearing high-binding peptides (#8, #17, and #23) accumulated predominantly on the surface of cancer cells. In contrast, sEVs with low-binding peptides (#7, #21, and #22) showed no detectable external signals, although weak RFP signals were observed intracellularly. These findings suggest that high-binding scrambled peptides enhance the binding of engineered sEVs to cancer cell surfaces but do not promote effective internalization, resulting in sEV retention on the cell surface rather than facilitating endocytosis.

**Figure 2:**
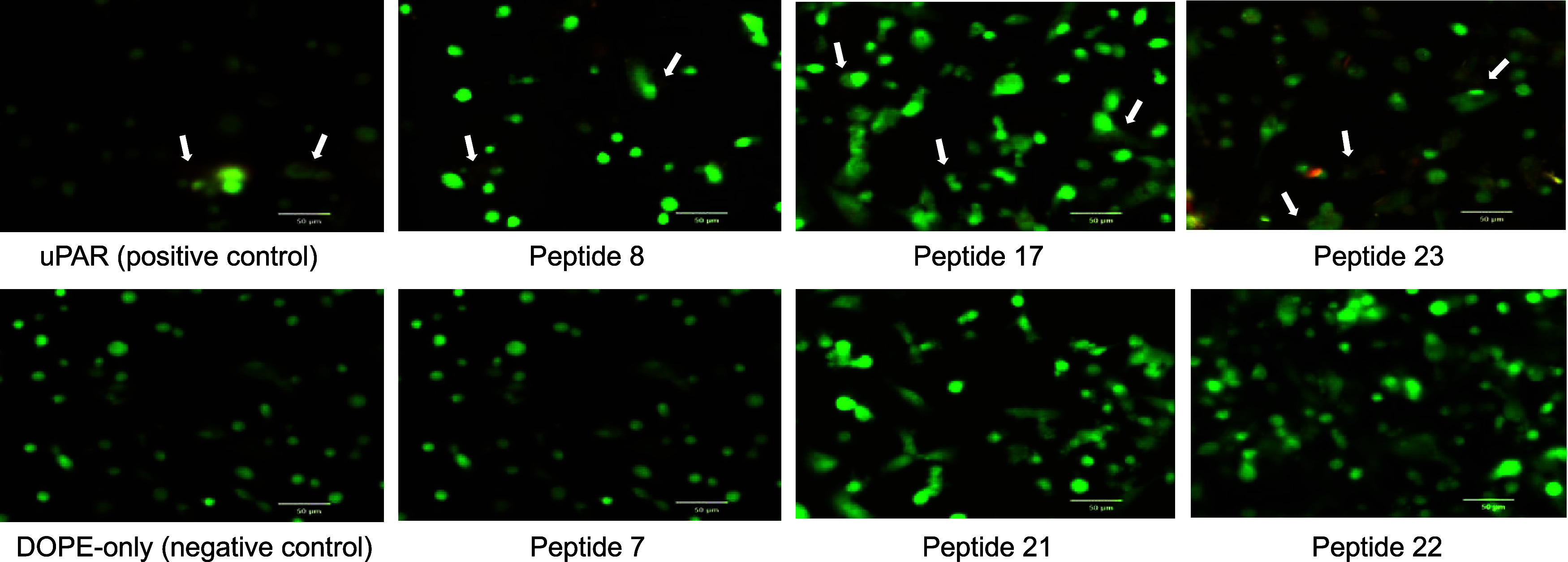
Fluorescence microscopy reveals differential cell surface attachment of PalmReNL-sEVs engineered with high- and low-binding scrambled uPAR-binding peptides. MDA-MB-231 cells were incubated with PalmReNL-sEVs (red) displaying scrambled peptides identified from bioluminescence screening. High-binding peptides (#8, #17, and #23) and low-binding peptides (#7, #21, and #22) were compared. Following 3-h incubation with the engineered PalmReNL-sEVs, MDA-MB-231 cells were stained with CellTrace-CFSE (green). Merged fluorescent images were shown. Scale bars, 50 μm. Arrows indicate extracellularly accumulated PalmReNL-sEVs.

### Accelerated sEV Production and Methotrexate (MTX) Loading via Cell Scraping

MTX, a widely used chemotherapy drug, inhibits dihydrofolate reductase (DHFR), thereby disrupting DNA synthesis and cell proliferation.^30^ However, MTX resistance in certain cancer cells significantly limits its therapeutic efficacy, necessitating the development of novel drug delivery systems to overcome this resistance. Such approaches include albumin-conjugated MTX,^31^ fibrinogen-MTX,^32^ lipoamino acid-MTX,^33^ and cell-penetrating peptide-MTX conjugates.^34^ Notably, the triple-negative breast cancer cell line MDA-MB-231 exhibits MTX resistance due to reduced expression of the folate carrier protein essential for drug uptake.^35^

To establish baseline MTX resistance, we treated MDA-MB-231 cells with varying concentrations of MTX for 3 h, followed by washing and a 48-h recovery period. MTX concentration was quantified by measuring absorbance at 313 nm as previously reported,^36^ with absorbance values showing a linear relationship with MTX concentration (Figure S5A). Cell viability was assessed through nuclear staining and counting, a method previously validated for accurate evaluation.^37^ As expected, MDA-MB-231 cells showed substantial drug resistance, with cell viability decreasing by only 43.3% even at the highest tested MTX concentration of 500 μM (Figure S5B,C). Notably, this plateau in cell death remained constant across the concentration range. We therefore used this MTX-resistant model system to evaluate whether peptide-engineered sEVs could enhance drug uptake in MDA-MB-231 cells and overcome the limited therapeutic response observed with conventional MTX treatment.

sEVs can be loaded with therapeutic cargo through either passive or active methods to serve as drug delivery nanocarriers.^38^ Passive loading relies on the natural incorporation of drugs into sEVs during cellular drug exposure, making it particularly suitable for membrane-permeable hydrophobic compounds. While straightforward to implement, passive loading often results in suboptimal drug encapsulation. In contrast, active loading methods utilize physical or chemical interventions, such as electroporation, sonication, or freeze–thaw cycles, to facilitate drug entry into sEVs.^17,38^ These techniques can achieve higher loading efficiencies but may affect sEV integrity and functionality.

Mechanical stress has been shown to enhance sEV release, likely through plasma membrane repair after wounding with calcium signaling.^29,39,40^ We therefore investigated whether physical agitation via cell scraping could simultaneously enhance both sEV yield and drug loading when treating cells with MTX. We confirmed that cell scraping rapidly induces plasma membrane damage, as evidenced by the efficient uptake of FITC-dextran, a membrane-impermeable molecule, into HEK293FT cells. In contrast, control cells without scraping showed no detectable intracellular fluorescence, consistent with previous reports^41^ (Figure S6). While this approach could rapidly produce drug-loaded sEVs, a potential downside might be the production of dramatically increased numbers of sEVs without adequate drug incorporation. To test this hypothesis, HEK293FT cells were treated with 2 mM MTX for 3 h, then either scraped or left unagitated before supernatant collection. The sEV fractions were purified from the collected supernatants and characterized using NTA. Of note, only short-term MTX treatment even without scraping produced three times more sEVs compared to the number produced under 48-h culture in EV-depleted medium (Figure 3A). Similar upregulation of sEV production has been previously observed under various chemotherapy drug treatments.^42,43^ Cell viability following 2 mM MTX treatment of HEK293FT cells for 3 h was 91.5% ± 4.7% (mean ± SD, *n* = 4), compared to 100% viability in untreated control cells, indicating only modest cytotoxicity under these conditions. Moreover, cell scraping after MTX treatment significantly increased sEV yield, producing 1.4 × 10^11^ EVs/mL compared to 6.3 × 10^10^ EVs/mL without scraping—a 2.2-fold increase. The mean diameters of sEVs were comparable between conditions: 128.5 nm with scraping and 135.8 nm without scraping (Figure 3A). MTX loading into sEVs was further evaluated by incubating EV producer cells with varying concentrations of MTX, followed by cell scraping. The results demonstrated that MTX loading efficiency was concentration-dependent, and that cell scraping increased the total amount of MTX carried in sEVs (Figure S7). Notably, at the highest MTX concentration (6 mM), the total MTX content in sEVs was comparable with or without scraping, despite a 1.9-fold increase in sEV production. This may be attributed to cytotoxic effects of 6 mM MTX or the high DMSO concentration (10.8%) used during treatment. The MTX-loaded sEVs under the 2 mM MTX condition were further characterized by Western blot analysis to detect the sEV marker tetraspanins CD63 and CD81. Control sEVs were isolated from conditioned medium of HEK293FT cells cultured in EV-depleted media for 48 h. Following 3-h 2 mM MTX treatment, sEV fractions from both scraped and unscraped cells showed markedly elevated CD81 levels. However, CD63 expression was notably reduced in these rapidly produced sEVs compared to controls (Figures 3B and S8). CD63 is predominantly associated with endosome-derived exosomes, while CD81 is primarily found in small ectosomes budding from the plasma membrane.^44^ This differential expression pattern—high CD81 and low CD63—suggests that MTX treatment may selectively enhances the release of plasma membrane-derived sEVs while minimally affecting endosomal vesicle production, regardless of cell scraping. Transmission Electron Microscopy (TEM) further confirmed the presence of characteristic cup-shaped vesicles in these rapidly produced sEVs from both scraped and unscraped conditions (Figure 3C). Importantly, we observed similar sample purity between MTX-treated and untreated conditions with no evidence of MTX crystallization, ruling out potential artifacts from MTX nanoparticle contamination.

**Figure 3:**
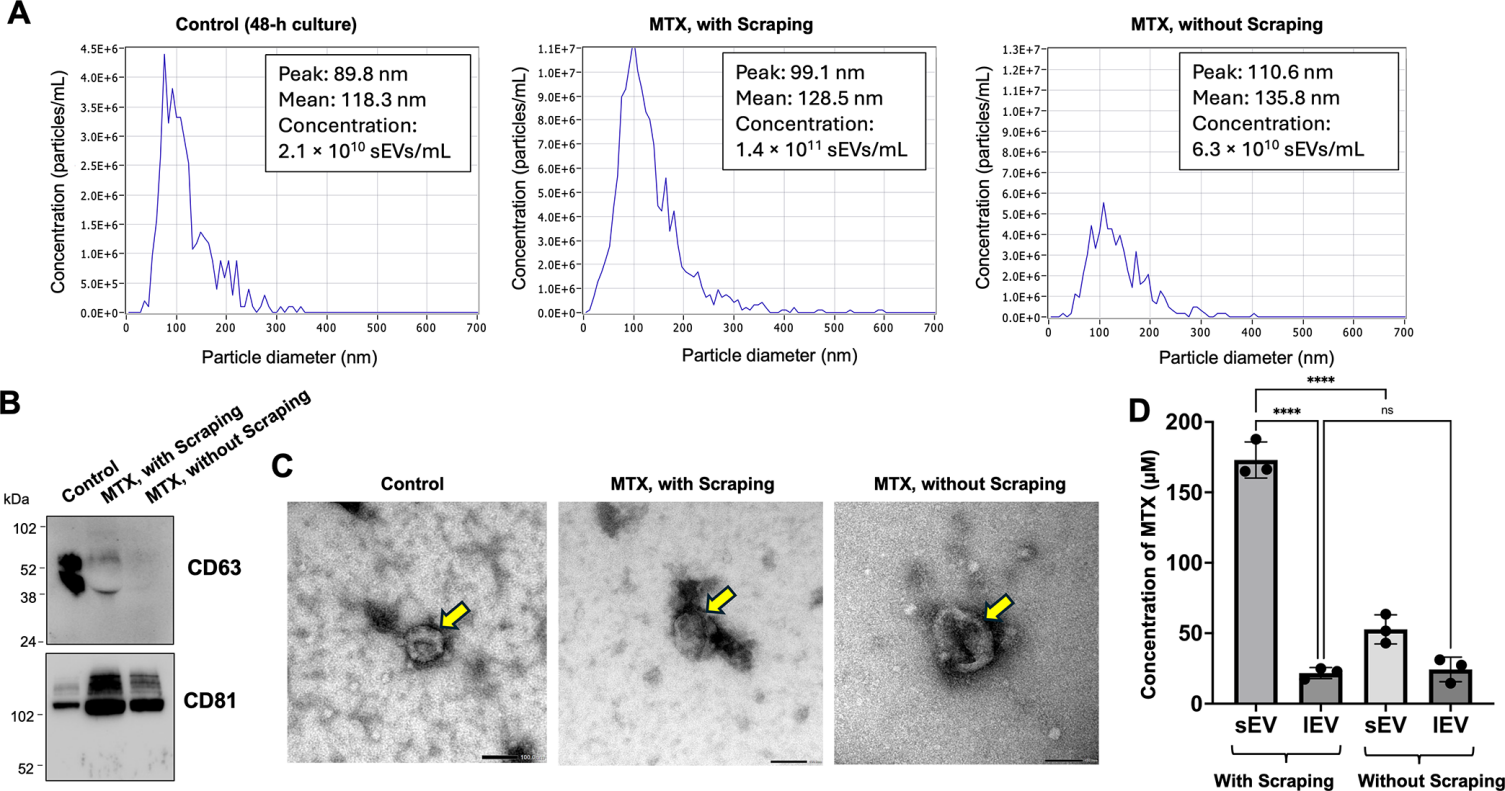
Cell scraping accelerates the production of MTX-loaded sEVs. (A) NTA of sEVs released from HEK293FT cells under conventional 48-h production without MTX (Control) or 2-h rapid production with MTX (2 mM), with or without scraping. Size distribution histograms show particle concentration per size interval (particles/mL). The particle concentration, peak size, and mean size are indicated in the figure. (B) Western blot analysis of EV markers CD63 and CD81 in sEVs isolated under different conditions. Equal volumes of sEV samples (15 μL) purified from 2 × 10^6^ cells were loaded to assess relative EV marker expression levels. (C) Representative TEM images of sEVs (yellow arrows) produced under different conditions. Scale bars, 100 nm. (D) Quantification of MTX loading in lEVs and sEVs following cell scraping-induced release. MTX concentrations in purified lEVs and sEVs were determined through spectrophotometric analysis. Error bars represent SD (*n* = 3).

While several studies have demonstrated MTX loading into large EVs (lEVs),^45,46^ a direct comparison of MTX loading efficiency between sEVs and lEVs has not been reported to the best of our knowledge. We compared MTX loading into rapidly produced sEVs and lEVs, with and without cell scraping (Figure 3D). Following the established protocol,^19,27^ we isolated lEVs by centrifugation at 20,000*g* for 30 min, then purified sEVs from the resulting supernatant using ultrafiltration. MTX quantification via absorbance at 313 nm revealed that scraped sEVs contained 6.8-fold higher MTX levels compared to lEVs. Furthermore, scraped sEVs showed 3.4-fold higher MTX concentrations than unscraped sEVs, while MTX loading in lEVs remained consistent regardless of scraping. These findings demonstrate that sEVs serve as more effective MTX delivery vehicles than lEVs and that cell scraping significantly enhances drug loading into sEVs.

### Peptide-Functionalized sEVs for Targeted MTX Delivery to Drug-Resistant Cancer Cells

Several scrambled THPs enhanced sEV uptake by cancer cells (Figure 1B), leading us to evaluate the therapeutic potential of MTX-loaded sEVs functionalized with these peptides. We assessed the cancer cell-killing effects of MTX-loaded sEVs modified with a low-binding peptide (#7), three high-binding peptides (#8, #17, and #23), and the original uPAR-binding peptide. MTX-loaded sEVs were rapidly generated by incubating HEK293FT cells for 3 h with 2 mM MTX, followed by cell scraping. Isolated sEVs were functionalized with DOPE-conjugated peptides (Figure 4A). Concentrations of MTX loaded in sEVs were determined based on absorbance at 313 nm. MDA-MB-231 cells were treated with either 250 μM of free MTX or equivalent doses of MTX loaded in peptide-functionalized sEVs for 3 h. Following PBS washes, cells were cultured for an additional 48 h in complete medium prior to assessing cell killing efficacy.

**Figure 4:**
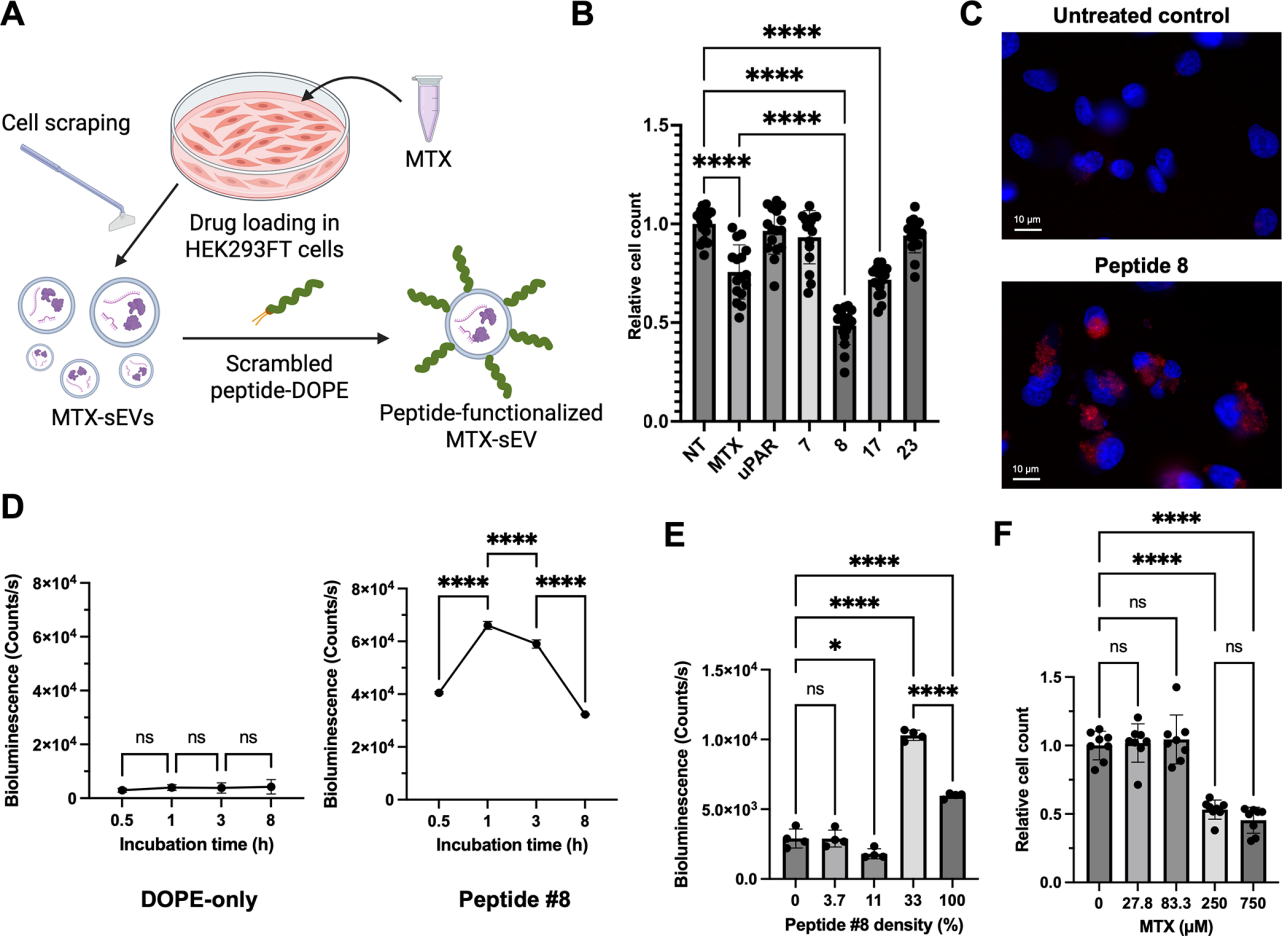
MTX-loaded sEVs functionalized with scrambled THPs exhibit enhanced cell killing in MDA-MB-231 cells. (A) Schematic representation of the production of MTX-loaded, peptide-functionalized sEVs. The EV producer cells were treated with 2 mM MTX for 3 h, followed by cell scraping for rapid EV production. Isolated sEVs were then functionalized with DOPE-conjugated scrambled peptides (Created with BioRender.com). (B) MDA-MB-231 cells (40,000 cells/well) were treated with MTX-loaded, peptide-functionalized sEVs (5.0 × 10^8^ particles/well) for 3 h, followed by a 48-h recovery period in fresh medium. Control groups included equivalent concentrations of free MTX (250 μM) and nontreated cells (NT). Following treatment, cells were fixed and nuclei were stained with Hoechst 33342 for quantification by nuclear counting. Cell viability is reported as relative cell numbers normalized to NT controls. Error bars represent SD (*n* = 16). (C) MDA-MB-231 cells were cultured with PalmReNL-sEVs (red) displaying Peptide #8 overnight. Cell nuclei were stained with Hoechst 33342 (blue). Scale bars, 10 μm. (D) MDA-MB-231 cells (40,000/well) were incubated with PalmReNL-sEVs (3.8 × 10^8^ particles/well) functionalized with either DOPE-only or DOPE-Peptide #8 for varying time points prior to bioluminescence measurement. (E) PalmReNL-sEVs functionalized with varying densities of DOPE-Peptide #8 were incubated with MDA-MB-231 cells for 3 h to evaluate cellular uptake efficiency. (F) MDA-MB-231 cells were treated with different doses of MTX-loaded sEVs functionalized with Peptide #8 for 3 h, followed by a 48-h recovery period to assess cytotoxic effects by nuclear counting..

Among the high-binding peptides, sEVs functionalized with Peptide #8 demonstrated reduced viability by 51.6%, while free MTX reduced viability by 24.4%, indicating a 2.1-fold greater cytotoxicity compared to free MTX. On the other hand, Peptides #17 and #23 exhibited comparable or reduced efficacy in intracellular MTX delivery (Figures 4B and S9). Interestingly, the original uPAR-binding peptide failed to significantly enhance cancer cell death when delivering MTX-loaded sEVs. These results suggest that drug delivery efficiency and subsequent cytotoxicity do not directly correlate with cancer cell binding affinity. Rather, the positioning of scrambled amino acids within the peptides likely plays a crucial role in mediating both cellular internalization of MTX-loaded sEVs and their therapeutic efficacy. As expected, PalmReNL-sEVs modified with Peptide #8 showed pronounced accumulation in the perinuclear regions after overnight incubation (Figure 4C), although nuclear import was not observed. It is worth mentioning, however, that this reporter system cannot distinguish between intact sEVs and those that have undergone endosomal fusion, limiting its ability to directly monitor intracellular drug release following sEV internalization. Uptake of functionalized PalmReNL-sEVs with Peptide #8 was further assessed at varying incubation time points (0.5, 1, 3, and 8 h) by measuring bioluminescence signals (Figure 4D). Unexpectedly, longer incubation times (3 and 8 h) resulted in lower signals compared to the 1-h time point. We previously demonstrated that NanoLuc within PalmReNL is sensitive to acidic intracellular environments, leading to reduced catalytic activity.^19^ This suggests that the functionalized sEVs were internalized by MDA-MB-231 cells and trafficked into acidic compartments, such as endosomes or lysosomes. Additionally, PalmReNL-sEVs were functionalized with varying concentrations of DOPE-Peptide #8 to assess the impact of peptide density on cancer cell uptake (Figure 4E). Interestingly, reducing the DOPE-Peptide #8 concentration to 33% of the original level resulted in 1.7-fold increase in uptake by MDA-MB-231 cells, suggesting that excessive peptide density on the sEV surface may hinder efficient cellular interaction. Finally, the therapeutic efficacy of Peptide #8-engineered sEVs loaded with MTX was evaluated by treating cells with varying doses (Figure 4F). Lower MTX doses delivered via sEVs did not significantly affect cancer cell viability, whereas a 3-fold higher doses induced notable cytotoxicity, resulting in 55% cell death in MDA-MB-231 cells. Further investigation into the correlation between specific amino acid positions and intracellular drug delivery efficacy via engineered sEVs is warranted in future studies.

In summary, this research presents a paradigm shift in developing THPs for sEV-based drug delivery systems. While amino acid scrambling has traditionally served as a method to create negative controls when investigating functional peptides, our study reveals that such scrambling can unexpectedly yield superior cancer cell targeting rather than diminishing functionality. Using the uPA-derived uPAR-binding peptide sequence, we constructed a scrambled peptide library to evaluate peptide-functionalized sEVs for cancer cell binding. Remarkably, we discovered that only specific amino acid positions within the scrambled peptides enhanced cancer cell targeting of sEVs, while other positions effectively functioned as negative controls. This finding suggests that high affinity to cancer cells depends not merely on amino acid composition, overall charge, or hydrophobicity, but also critically on sequence patterns. Similar phenomena have been observed with other peptides, including Bac2A (a short antimicrobial peptide derived from bovine bactenecin^47^ and the RNA-binding domain of yeast poly­(A)-binding protein (YPAB)).^48^ Sequence scrambling of these peptides produced variants with activities or binding affinities ranging from superior to equivalent to the original sequences.

Conventional approaches to designing THPs have focused solely on maximizing peptide affinity to cancer cells, assuming that effective binding to target molecules on cell surfaces enables peptides with payloads to undergo internalization via receptor-mediated or independent endocytosis.^49^ Our findings underscore the critical importance of amino acid patterns in facilitating intracellular drug delivery processes. While sEVs are generally considered promising drug delivery vehicles, their inherent heterogeneity poses significant challenges for clinical applications. This heterogeneity can lead to batch-to-batch variability in drug loading efficacy and surface ligand presence, making it difficult to achieve consistent therapeutic outcomes. Controlling these variables during the development of engineered sEVs remains a key obstacle for clinical applications. Our research demonstrates that peptide functionalization can enhance sEV-mediated drug delivery to resistant cancer cells. Notably, our findings suggest that higher cellular binding of sEVs does not necessarily correlate with higher drug delivery efficacy through cellular internalization processes. This finding highlights the need to optimize both surface interactions and cellular uptake mechanisms when designing targeted sEV therapeutics.

## Conclusions

Our results provide compelling evidence for future investigation aimed at optimizing peptide amino acid sequences. Specifically, THPs can be further enhanced through strategic scrambling to achieve more efficient drug delivery via engineered sEVs. These insights have significant implications for advancing our understanding of complex peptide-based drug delivery mechanisms and may lead to the development of more effective targeted therapies.

## Material and Methods

### Cell Lines and Cell Culture

HEK293FT (R700–07, Invitrogen) and MDA-MB-231 cells (ATCC) were cultured in Dulbecco’s Modified Eagle Medium (DMEM) supplemented with 10% (v/v) fetal bovine serum and 1% penicillin/streptomycin (Gibco, 15070063). HEK293FT cells stably expressing PalmReNL were maintained with 2 μg/mL puromycin selection. All cells were incubated at 37 °C in a 5% CO_2_ atmosphere.

### Plasmid DNA Construction

For stable expression of the EV reporter PalmReNL, we previously developed a Sleeping Beauty transposon vector,^50^ pKT2/CAGXSP/PalmReNL^19^ (available from Addgene, plasmid #182970).

### Isolation of sEVs

EV-depleted FBS was prepared by 18-h ultracentrifugation at 100,000*g* at 4 °C.^51^ HEK293FT and PalmReNL-HEK293FT cells were plated at 2 × 10^6^ cells per 100 mm cell culture dish with EV-depleted media and cultured for 48–72 h until reaching sufficient confluency. The conditioned media were centrifuged at 600*g* for 5 min to remove cells and debris. The supernatants were then filtered through 0.2 μm PES membrane filters (Nalgene, 725–2520) to remove large vesicles. sEVs were enriched using a size-based isolation method with 50 nm porous membranes (Whatman, WHA110603) and holders (EMD Millipore, SX0002500) by applying vacuum pressure.^19^ The sEVs on the membranes were washed with PBS and carefully collected. After purification, a 10% freezing solution containing 250 mM trehalose was added prior to storage at −80 °C.

### Nanoparticle Tracking Analysis (NTA)

The size and number of sEVs were characterized using NTA (ZetaView, Particle Metrix). Prior to analysis, isolated sEVs were diluted in PBS to achieve an optimal particle concentration (1 × 10^9^–1 × 10^10^ particles/mL). Automated measurements were taken at 11 distinct positions in the sample cell. The instrument’s outlier control feature selected high-quality videos for analysis. Particle size distribution (nm) and concentration (particles/mL) were calculated based on the principles of Brownian motion and light scattering.

### Membrane Functionalization of sEVs with Peptides

Twenty-four scrambled amino acid sequences were designed through sequence permutation of the uPAR-binding peptide (GenScript, Piscataway, NJ). DOPE-NHS (dioleoylphosphatidylethanolamine *N*-hydroxysuccinimide; COATSOME FE-8181SU5, NOF America, White Plains, NY) was coupled to uPAR-binding, scrambled, and nonspecific peptides (GenScript, Piscataway, NJ) for self-insertion of the peptide-DOPE molecules into the membrane of sEVs as previously demonstrated.^25^ Briefly, peptides (50 μg) and DOPE-NHS (1.25 μg) were dissolved in DMSO and combined with a 20-fold molar excess of peptides in the presence of an equal vol of HEPES buffer (25 mM, pH 7.5), allowing the mixture to react at room temperature for 1 h to form the DOPE-peptides. Then, 10% (v/v) Tris buffer (100 mM, pH 7.5) was added to stop the reaction. A negative control, DOPE-only, was prepared in the same condition without peptides. PalmReNL-sEVs (1.5 × 10^9^) were then added and incubated with DOPE-THPs or DOPE only at 37 °C for 1 h. The engineered sEVs were concentrated using 100 kDa ultrafiltration spin filters (UFC810024, MilliporeSigma, Burlington, MA) and washed with PBS twice by centrifugation. A 10% freezing solution containing 250 mM trehalose was added to the engineered sEVs prior to storage at −80 °C. Dissociation constants (*K*_d_) of peptide-functionalized PalmReNL-sEVs in uptake by MDA-MB-231 cells were calculated using Origin 9 (OriginLab Corporation, Northampton, MA).

To quantify the incorporation of THPs on sEVs, a fluorescently labeled uPAR-binding peptide (VSNKYFSNIHWG-Lys­(FITC)-C) was synthesized (GenScript). These peptides were conjugated to DOPE-NHS and incubated with sEVs (1.5 × 10^9^) in the same condition. After washing the engineered sEVs with PBS, THP incorporation was quantified by measuring fluorescence intensity. Inserting efficacy was calculated as the ratio of fluorescence intensity from the engineered sEVs to that of the initial fluorescent peptides. The conjugation efficiency of THPs to sEVs via the DOPE-NHS linker was determined to be 6.1 ± 1.9% (mean ± SD, *n* = 4), based on this fluorescence ratio.

### Fluorescence Microscopy and Bioluminescence Measurement

The uptake of PalmReNL-sEVs by MDA-MB-231 cells was analyzed by fluorescence microscopy and bioluminescence measurement. The cells were plated in black or clear 96-well cell culture plates at a concentration of 40,000 cells/well and cultured for 24 h before adding the peptide-functionalized PalmReNL-sEVs (3.8 × 10^8^ particles/well). After 3-h incubation, the cells were washed twice with PBS and the uptake of reporter sEVs was analyzed by measuring quantitative bioluminescence signals from reporter sEVs taken up by MDA-MB-231 cells in each well after adding furimazine (Fz; 25 μM) using a Spark Multimode Microplate Reader (Tecan, Männedorf, Switzerland). For qualitative assessment of cellular sEV uptake at the single-cell level by fluorescence microscopy, MDA-MB-231 cells were stained with CellTrace CFSE (C34554, Invitrogen, Waltham, MA) or Hoechst 33342 (H3570, Life Technologies), and the uptake of engineered PalmReNL-sEVs was characterized using a BZ-X700 fluorescence microscope (KEYENCE, Osaka, Japan). All images were further analyzed using ImageJ software (NIH, Bethesda, MD). To visualize individual PalmReNL-sEVs, a drop of the isolated sEVs was placed on hydrophobic PTFE printed slides (Electron Microscopy Sciences, 63429–04), as previously described.^29,52^ After 30 min of incubation at 4 °C, slides were washed twice with PBS and imaged using a Leica Thunder widefield fluorescence microscope with a 63× oil immersion objective lens (Leica Microsystems, Wetzlar, Germany). Image analysis was conducted using ImageJ software.

### Western Blotting

The isolated sEVs were lysed with 4X sample buffer (Bio-Rad, 1610747) without β-mercaptoethanol and heated at 75 °C for 5 min. To detect CD81 and CD63, samples were run on a 4–20% Mini-PROTEAN TGX Stain Free gel (Bio-Rad, 4568096) and transferred to PVDF membranes (Millipore, IPFL00010). Membranes were blocked with PBS containing 5% skim milk and 0.05% Tween20 (v/v) for 30 min at room temperature; incubated with primary antibodies overnight at 4 °C at dilutions recommended by the suppliers as follows: anti-CD81 (1:1,000; Proteintech, 66866), anti-CD63 (1:1,000; Ts63, Thermo Fisher, 10628D). Membranes were washed 3 times with PBS containing 0.05% Tween20 (v/v), incubated with HRP-conjugated antimouse IgG (1:10,000; Cell Signaling, 7076) for 1 h at room temperature, and washed again to remove unbound antibodies. Membranes were visualized with ECL Select Western Blotting Detection Reagent (GE Healthcare, RPN2235) on ChemiDoc MP Imaging System (Bio-Rad).

### Transmission Electron Microscopy

Isolated sEVs were fixed in 1% paraformaldehyde at room temperature for 30 min. A Formvar-coated copper grid was kept in a saturated water environment for 24 h and then placed on a 50 μL aliquot of sEV solution for 20 min incubation. Samples were washed by placing each grit face down on six consecutive 100 μL droplets of water. The samples were negative stained with 0.4% uranyl acetate and 1.8% methylcellulose embedding solution. Excess uranyl acetate was removed by contacting the grid edge with filter paper, followed by air-drying. Samples were observed using a JEOL 1400 Flash Transmission Electron Microscope equipped with an integrated Matataki Flash sCMOS bottom-mounted camera, operated at 100 kV.

### Spectrophotometric Quantification of Methotrexate (MTX)

MTX concentration was determined through spectrophotometric analysis using a Spark Multimode Microplate Reader (Tecan, Männedorf, Switzerland). A series of MTX solutions were prepared by serial dilution in DMEM culture medium. The specific absorption wavelength for MTX was identified at 313 nm through comprehensive spectral scanning. A linear correlation between MTX concentration and absorbance was established to enable accurate measurement of free MTX or EV-loaded MTX concentrations.

### Cell Scraping-Facilitated MTX Loading into sEVs

HEK293FT cells (2 × 10^6^ cells) were plated in 100 mm cell culture dishes and cultured for 24–48 h until reaching over 90% confluency. MTX was dissolved in DMSO at 55 mM to prepare a stock solution. The culture medium was removed, and cells were washed with 10 mL PBS, followed by treatment with 1 mL of MTX at final concentrations of 0.22, 0.67, 2, or 6 mM in DMEM (containing 0.4%, 1.2%, 3.6%, or 10.8% DMSO, respectively). Cell viability of EV producer HEK293FT cells was assessed using the trypan blue exclusion assay with a TC20 Automated Cell Counter (Bio-Rad). The cells were incubated for 3 h at 37 °C, and then the supernatant was collected in a 2.0 mL polypropylene tube with or without cell scraping. The collected cell suspension was incubated for an additional 1 h at 37 °C before isolating MTX-loaded EVs. For separating large EVs (lEVs) and sEVs, the sample was first centrifuged at 2,000*g* for 30 min to remove cells and debris. The supernatant was collected and then centrifuged at 20,000*g* for 30 min at 4 °C to isolate the more dense lEVs. The lEV pellet was washed twice with PBS by resuspension and centrifugation at 20,000*g* for 30 min at 4 °C. The remaining supernatant was processed for sEV enrichment using a 10 kDa ultrafiltration spin filter (UFC801024, MilliporeSigma, Burlington, MA), followed by two PBS washes by adding 3 mL PBS each time in the same filter. A 10% freezing solution containing 250 mM trehalose was added to the isolated lEVs and sEVs prior to storage at −80 °C.

To assess cell membrane damage caused by scraping, HEK293FT cells were treated with 20 μg/mL FITC-dextran (46945, MilliporeSigma) and then scraped. Cells were incubated for 10 min to allow uptake of FITC-dextran, followed by the addition of complete DMEM. The cells were then cultured for an additional 3 h to allow readhesion. Intracellular FITC-dextran uptake was evaluated using fluorescence microscopy.

### Determination of Cell Viability by Nuclei Counting

To ensure accurate analysis of cell viability, nuclei enumeration was conducted.^37^ After treatments, MDA-MB-231 cells were fixed in 1% paraformaldehyde at room temperature for 30 min and subsequently stained with 10 μg/mL Hoechst 33342 (H3570, Life Technologies) for fluorescence microscopy using a BZ-X700 microscope (KEYENCE, Osaka, Japan). Quantitative cell counting was performed by systematically analyzing all images using ImageJ software (NIH, Bethesda, MD).

### Statistical Analyses

All statistical analyses were performed using GraphPad Prism version 10 (GraphPad Software, Inc.). For all statistical tests, a p-value <0.05 was considered statistically significant. Comparisons between the two groups were conducted using an unpaired two-tailed Student’s *t* test. For experiments involving comparisons among more than two groups, a one-way analysis of variance (ANOVA) was performed, followed by Tukey’s posthoc test for multiple comparisons. All experiments were conducted with at least three biological replicates, and statistical analysis was performed on data from independent experiments.

## Supplementary Material



## Notes

Characterization of PalmReNL-sEVs; analysis of peptide library features for sEV functionalization; comparative evaluation of cellular uptake efficiencies of sEVs functionalized with scrambled uPAR-binding peptides; fluorescence microscopy of peptide-functionalized PalmReNL-sEV uptake; assessment of the limited MTX sensitivity of MDA-MB-231 cells; evaluation of plasma-membrane damage induced by cell scraping; characterization of MTX loading into sEVs; Western blot analysis of sEVs derived from MTX-treated cells with or without scraping; and fluorescence microscopy of MDA-MB-231 cells treated with MTX-loaded sEVs bearing scrambled peptides (PDF)
