# Supplementary material for: Engineered Peptide Scrambling for Enhanced Drug Delivery to Resistant Breast Cancer Cells via Small Extracellular Vesicles
Source: ACS Appl Bio Mater. 2025 Dec 1;8(12):10610–9. doi: 10.1021/acsabm.5c00582 (PMC12709616; doi:10.1021/acsabm.5c00582)
Supplement: Supplementary file 1 [file mt5c00582_si_001.pdf]

SUPPORTING INFORMATION

Title:
Engineered Peptide Scrambling for Enhanced Drug Delivery to Resistant Breast Cancer Cells via Small Extracellular Vesicles

Authors:
Anika Babel[1,2,3], Joe Yuan[3], Najla A. Saleh[1], Aimen Al-Hilfi[3,4], Sadhana Kilangodi[1,2], Jake Sun[5], Lelti Asgedom[5], Alicia Withrow[6], Assaf A. Gilad[3,4,7,8,9,*], Masamitsu Kanada[1,8,10,*]

Affiliations:
[1] Institute for Quantitative Health Science and Engineering (IQ), Michigan State University, East Lansing, Michigan, USA;
Emails: babelani@msu.edu (A.B.), adelsale@msu.edu (N.A.S.), kilangod@msu.edu (S.K.), kanadama@msu.edu (M.K.)
[2] College of Natural Science, Michigan State University, East Lansing, Michigan, USA;
Email: babelani@msu.edu (A.B.), kilangod@msu.edu (S.K.)
[3] Department of Chemical Engineering & Materials Science, Michigan State University, East Lansing, Michigan, USA;
Emails: babelani@msu.edu (A.B.), yuanjoe@msu.edu (J.Y.), alhilfia@msu.edu (A.A.), gilad@msu.edu (A.A.G.)
[4] College of Engineering, Michigan State University, East Lansing, Michigan, USA;
Email: alhilfia@msu.edu (A.A.), gilad@msu.edu (A.A.G.)
[5] College of Osteopathic Medicine, Michigan State University, East Lansing, Michigan, USA;
Email: sunjake@msu.edu (J.S.), asgedoml@msu.edu (L.A.)
[6] Center for Advanced Microscopy, Michigan State University, East Lansing, Michigan, USA;
Email: pastorle@msu.edu (A.W.)
[7] Department of Radiology, Michigan State University, East Lansing, Michigan, USA;
Email: gilad@msu.edu (A.A.G.)
[8] College of Human Medicine, Michigan State University, East Lansing, Michigan, USA;
Emails: gilad@msu.edu (A.A.G.), kanadama@msu.edu (M.K.)
[9] The Scojen Institute for Synthetic Biology, Reichman University, Israel;
Email: gilad@msu.edu (A.A.G.)
[10] Department of Pharmacology & Toxicology, Michigan State University, East Lansing, Michigan, USA;
Email: kanadama@msu.edu (M.K.)
[*] Authors to whom correspondence should be addressed.
Emails: gilad@msu.edu (A.A.G.), kanadama@msu.edu (M.K.)

Contents:
Figure S1-S9

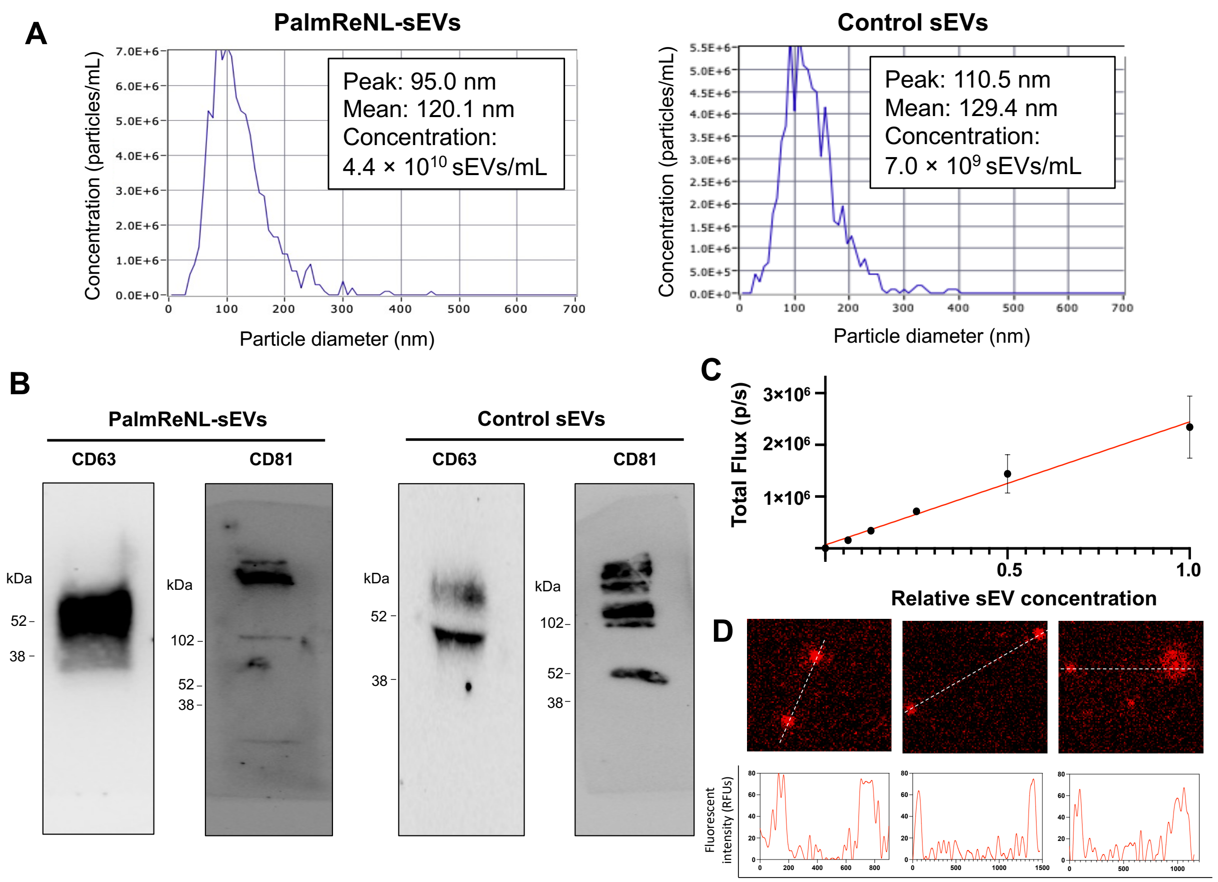

Figure S1. Characterization of PalmReNL-containing sEVs using bioluminescence assays and fluorescence microscopy. A) NTA comparing sEVs derived from PalmReNL-expressing HEK293FT cells (left) and untransfected control cells (right). **B)** Western blot analysis of EV markers CD63 and CD81 in PalmReNL-sEVs and control sEVs under non-reducing conditions. Equal protein amounts (15.8 µg) were loaded to assess marker expression. **C)** Bioluminescence quantification of PalmReNL-sEVs. **D)** Fluorescence microscopy detection of individual PalmReNL-sEVs, with corresponding fluorescence intensity line profiles. RFU, relative fluorescence units.

uPAR-binding peptide: VSNKYFSNIHWGC

		Charge	Hydrophobicity
1	NVIFGCWYSSHNK	1	-0.69
2	NVWCGFSHKISNY	1	-0.69
3	CHNVNFYWKIGSS	1	-0.69
4	FSIVYKNCGSNWH	1	-0.69
5	IWVSYKCFSNNGH	1	-0.69
6	IKNYCNSGWSHFV	1	-0.69
7	NSVWHCFKINSGY	1	-0.69
8	GVSCFISKYNNWH	1	-0.69
9	INHVSYWKNCSGF	1	-0.69
10	NCVYHGISFWKNS	1	-0.69
11	SHCVNFINYKGWS	1	-0.69
12	SNCYIFNSVGWHK	1	-0.69
13	KYSNFIGCWVSHN	1	-0.69
14	NCSSKFIVHGWYN	1	-0.69
15	YSISKHNWNGVFC	1	-0.69
16	NCYSWVIGFSNHK	1	-0.69
17	NSYGCSHIVFNWK	1	-0.69

		Charge	Hydrophobicity
18	NSHIKWVGCSYFN	1	-0.69
19	NICFSNHYGKVWS	1	-0.69
20	NVCIYNGSHKFWS	1	-0.69
21	SNCKWGVNHYFIS	1	-0.69
22	GISKYCFSNHVNW	1	-0.69
23	NSYKCINVSHWGF	1	-0.69
24	FWIYKHNCVSNGS	1	-0.69
25	WNWHECFTQHKQQ	0	-0.38
26	HKVSKHTQNDCFF	1	0.06
27	QQTGWSLFDSKKH	1	0.11
28	DHKQQNKSWGAII	1	0.15
29	NKCTNWCLDSWGE	-1	-0.1
30	KAFCNHKDINWGR	2	0.21
31	NKHDFCEVRENWK	0	0.73
32	TDKIVPSWGTKHC	1	0.02
33	FVFKHKWNCHKRE	3	0.25

Figure S2. Peptide library characteristics for sEV functionalization. Listed are the amino acid sequences, net charge, and hydrophobicity values for all peptides used in this study. Peptides 1-24: scrambled uPAR-binding peptides; Peptides 25-33: 13-mer non-specific peptides. Net charge and hydrophobicity values were calculated using the BACHEM Peptide Calculator.

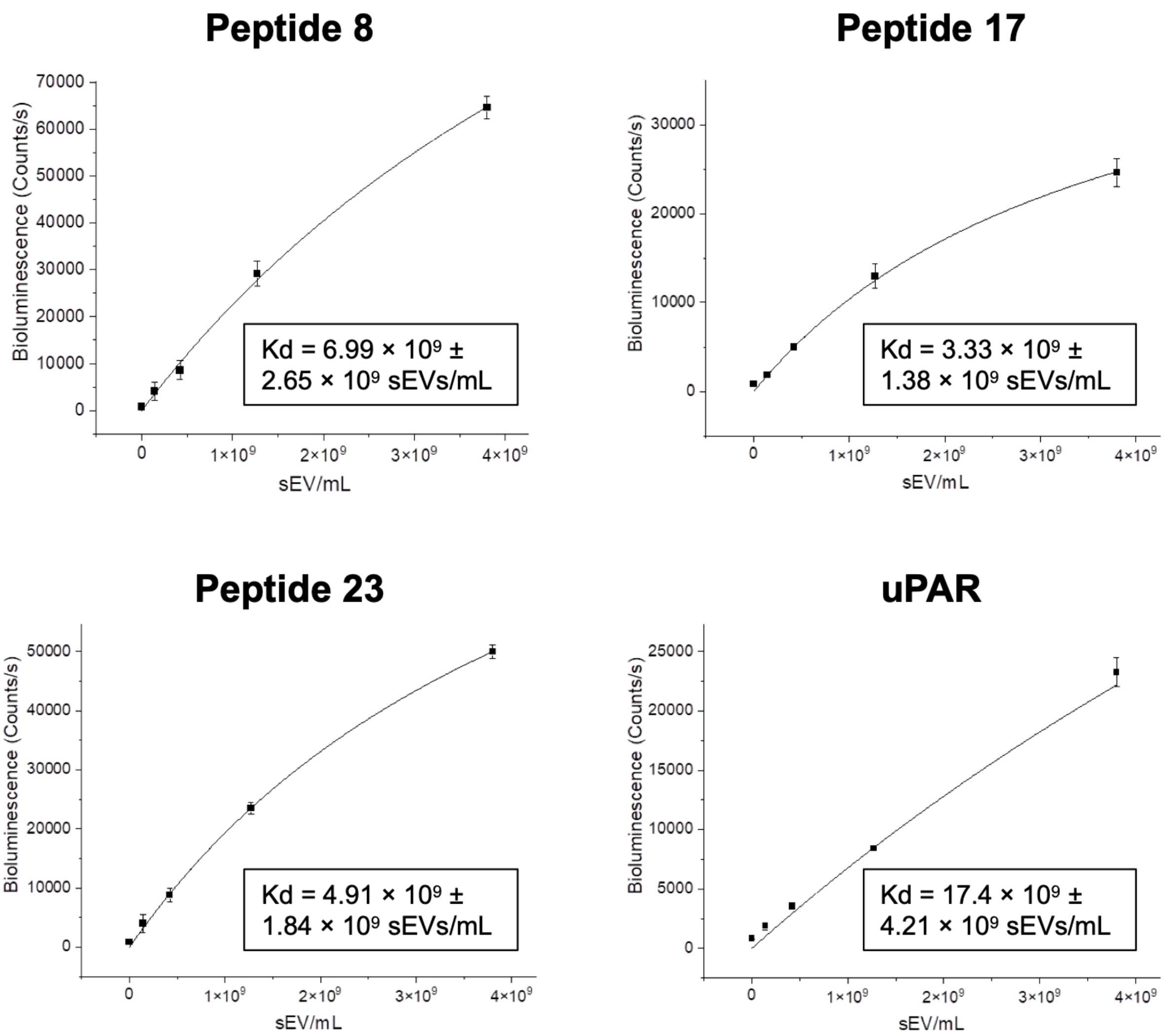

Figure S3. Comparative cellular uptake efficiencies of functionalized sEVs with scrambled uPAR-binding peptides. PalmReNL-sEVs were engineered with Peptides #8, #17, #23, or the original uPAR-binding peptide. Their cellular uptake was assessed using 3-fold dilutions from the original PalmReNL-sEV concentrations (3.8×10^9 particles/mL) following 3-h incubation with MDA-MB-231 cells (40,000/well). Kd values of these functionalized sEVs were calculated using Origin 9 software. Error bars represent SD (n = 4).

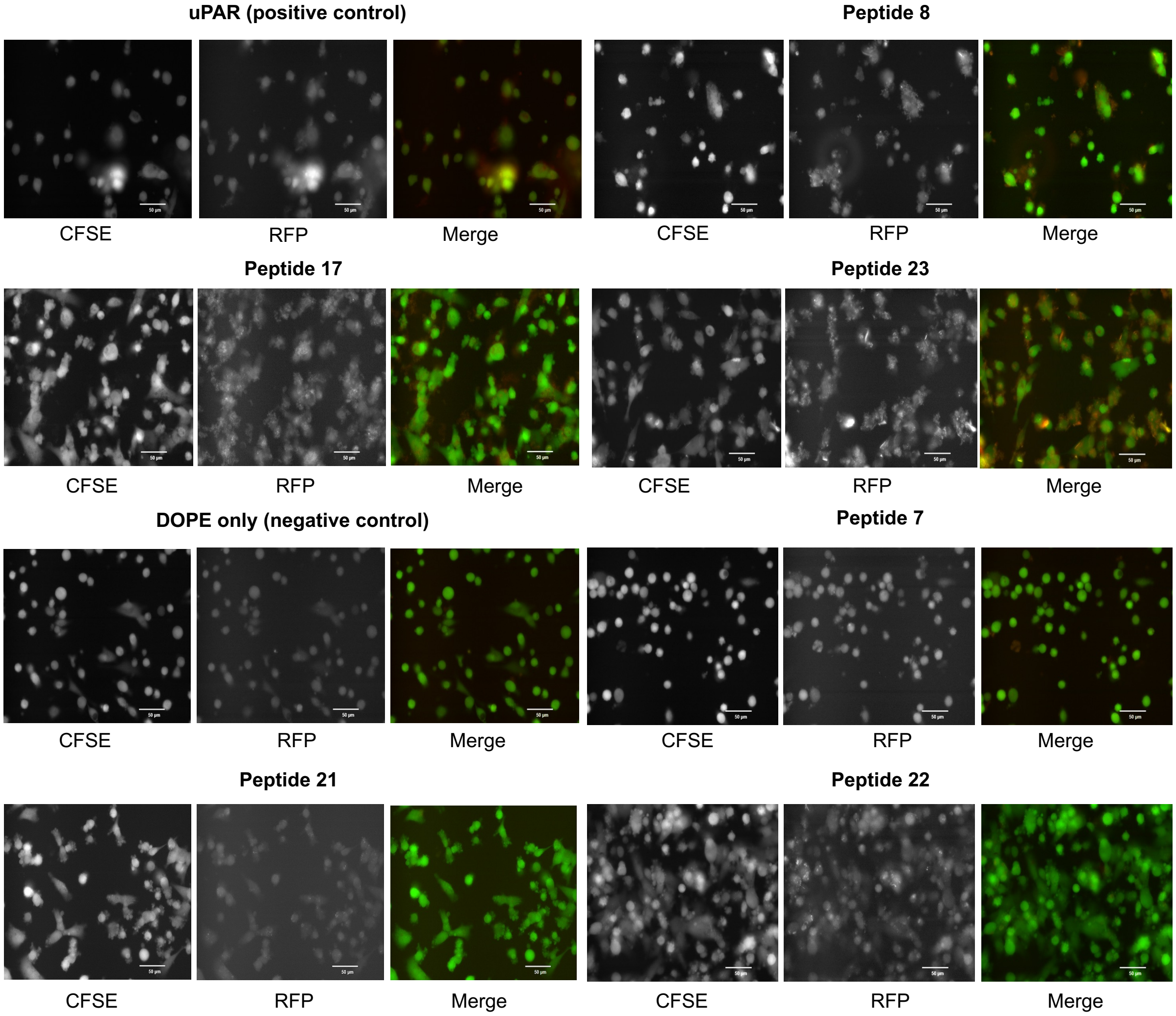

Figure S4. Fluorescence microscopy images of peptide-functionalized PalmReNL-sEVs and MDA-MB-231 cells. Cells were incubated with PalmReNL-sEVs (red) displaying high-binding peptides (8, 17, and 23) or low-binding peptides (7, 21, and 22). MDA-MB-231 cells were stained with CellTrace-CFSE (green). Scale bars, 50 μm.

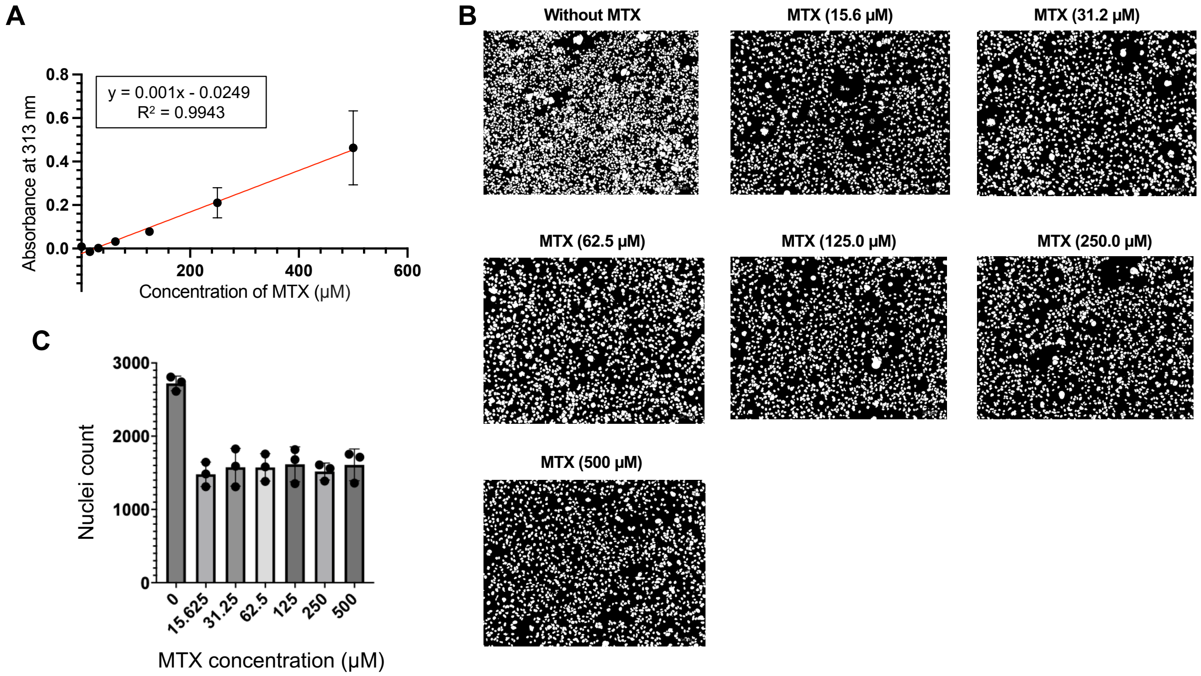

Figure S5. MDA-MB-231 cells show limited sensitivity to MTX treatment. A) MTX concentration in DMEM quantified by absorbance measurements at 313 nm. Error bars represent SD (n = 4). **B)** Representative fluorescence microscopy images of cells treated with increasing MTX concentrations for 3 h, followed by a 48-h recovery period. Non-viable cells were eliminated, and nuclei were stained with Hoechst 33342 (blue) for quantification. Scale bars, 50 µm. **C)** Quantitative analysis of cell nuclei following MTX treatments. Error bars represent SD (n = 3).

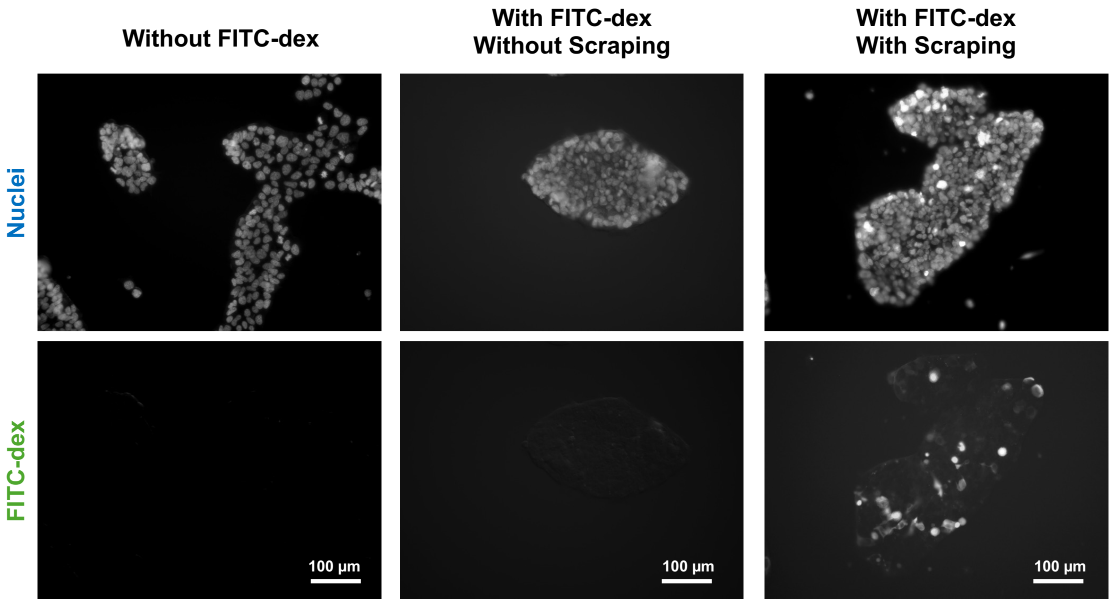

Figure S6. Cell scraping induced rapid and transient plasma membrane damage in HEK293FT cells. Cells were washed with PBS and treated with FITC-dextran (20 μg/mL in PBS), with or without scraping. Nuclei were stained with Hoechst 33342.

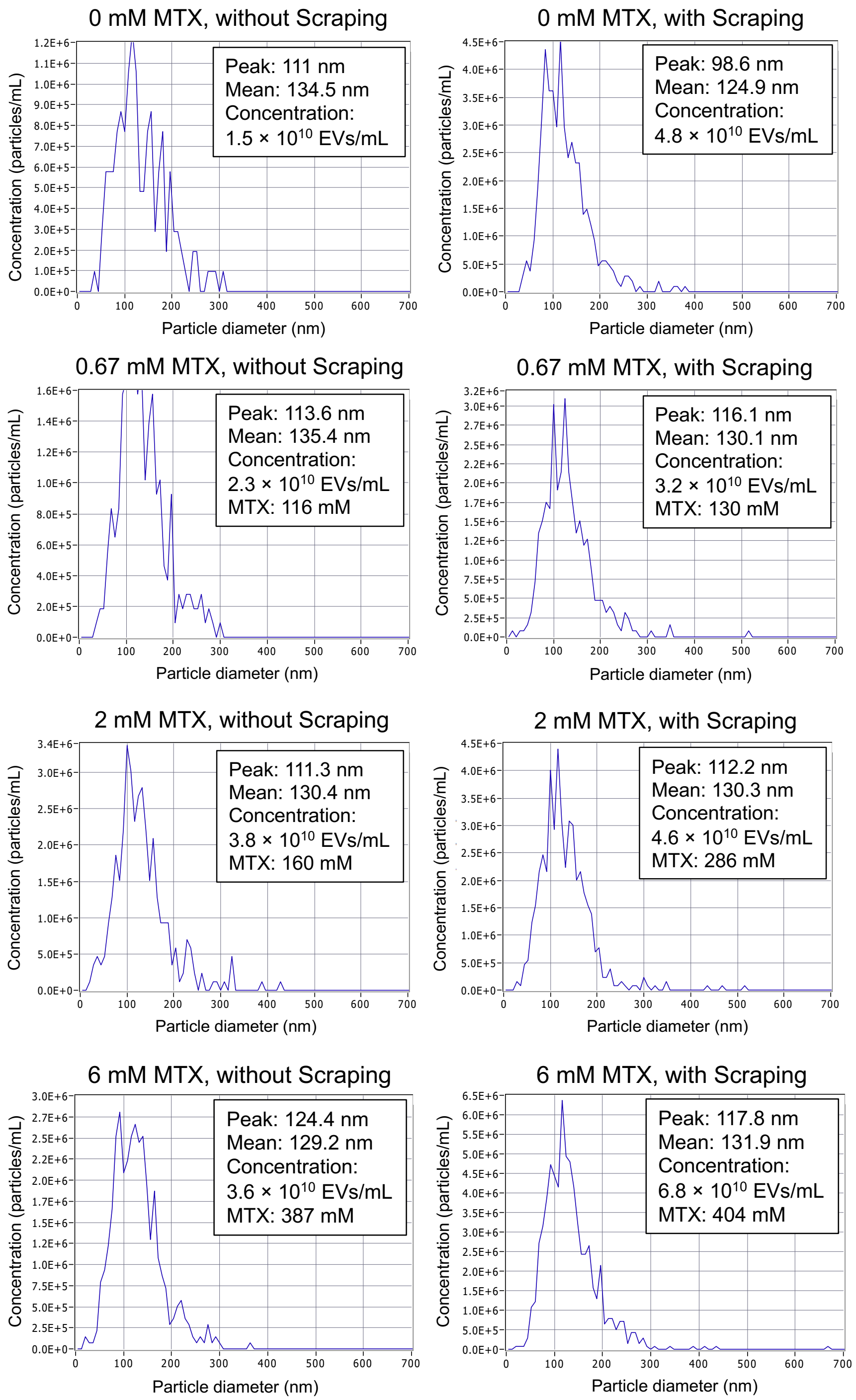

Figure S7. MTX loading into sEVs is concentration-dependent, while cell scraping increases MTX loading in sEVs. Cells were incubated with varying concentrations of MTX prior to scraping. After purifying sEVs from each supernatant, vesicle concentrations were analyzed by NTA, and MTX levels were quantified by absorbance at 313 nm.

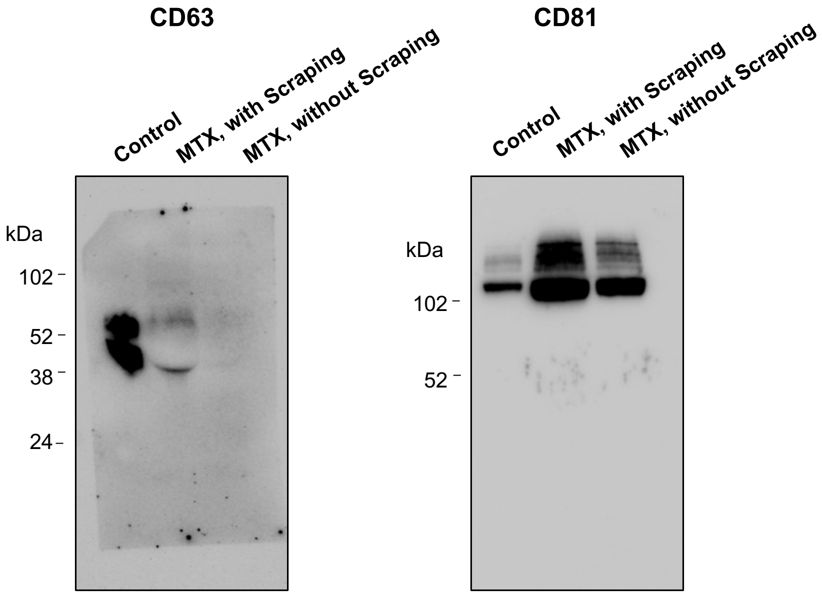

Figure S8. Western blot analysis of CD63 and CD81 proteins in sEVs derived from untreated control cells and cells treated with 2 mM MTX for 3 h, with or without scraping. Original images for Figure 3B.

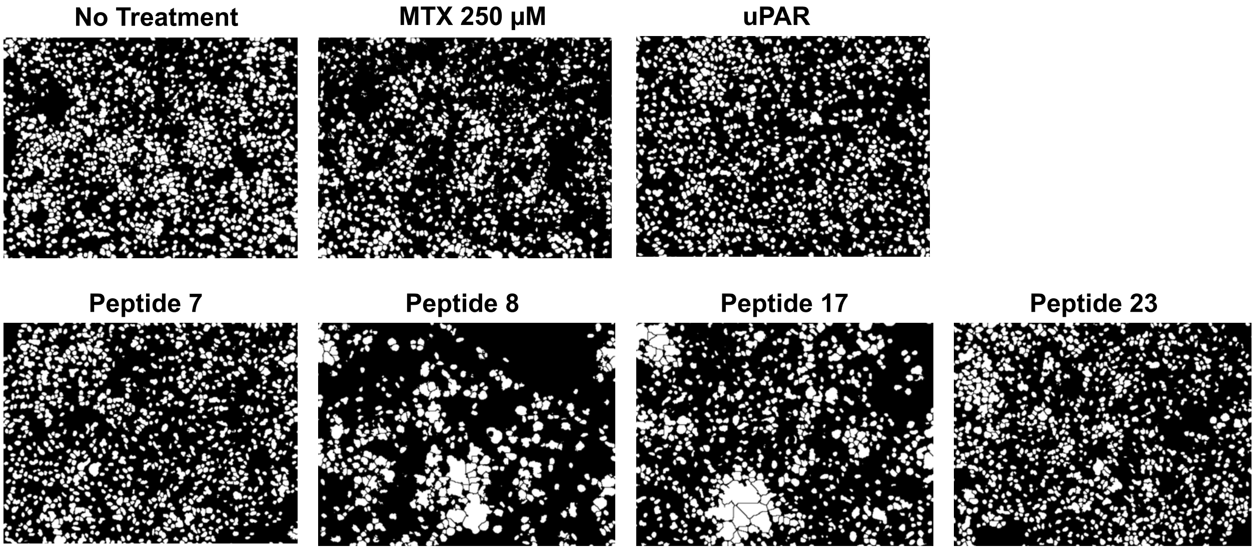

Figure S9. Representative fluorescence microscopy images of MDA-MB-231 cells treated with MTX-loaded sEVs functionalized with scrambled tumor-homing peptides. Cancer cells were treated with engineered sEVs for 3 h, followed by a 48-h recovery period. Non-viable cells were eliminated, and nuclei were stained with Hoechst 33342 for quantification.
